# A Sensorless Rotor Position Detection Method for Permanent Synchronous Motors Based on High-Frequency Square Wave Voltage Signal Injection

**DOI:** 10.3390/s26010028

**Published:** 2025-12-19

**Authors:** Anran Song, Zilong Feng, Bo Huang, Bowen Ning

**Affiliations:** 1 School of Mechanical and Electrical Engineering, Suqian University, Suqian 223800, China; 18129@squ.edu.cn; 2 Jiangsu Engineering Research Center of Key Technology for Intelligent Manufacturing Equipment, Suqian 223800, China; fengzilong@wust.edu.cn (Z.F.); 16638690961@163.com (B.H.); 3 Engineering Research Center for Metallurgical Automation and Measurement Technology of Ministry of Education, Wuhan University of Science and Technology, Wuhan 430081, China

**Keywords:** IPMSM, high-frequency square-wave injection, multiple coordinate systems, sixth-order quasi-proportional resonant, harmonic voltage compensation

## Abstract

**Highlights:**

**What are the main findings?**

- A sensorless control strategy for permanent magnet synchronous motors (PMSM) based on high-frequency square-wave injection with multi-coordinate transformation voltage harmonic suppression using a sixth-order quasi-proportional resonant (QPR) controller is proposed, which exhibits superior harmonic interference rejection performance and significantly reduced speed and torque fluctuations.
- The multi-coordinate transformation architecture can effectively extract the AC components of stator current harmonics by converting them into DC components.

**What are the implications of the main findings?**

- The establishment of the voltage compensation mathematical model facilitates the compensation of harmonic currents and enables real-time tracking of harmonic components.
- A sixth-order quasi-proportional resonant (QPR) controller, operating in parallel with the proportional-integral (PI) controller, can further suppress additional harmonics of the relevant order introduced during the compensation process.

**Abstract:**

To address the torque ripple and speed fluctuation issues in high-frequency square-wave injection-based sensorless control of interior permanent magnet synchronous motors (IPMSM) caused by low-order stator current harmonics (primarily the fifth and seventh), this paper proposes a harmonic voltage compensation strategy based on a sixth-order quasi-proportional resonant (QPR) controller, which effectively suppresses these specific harmonic disturbances. The proposed method, building upon conventional high-frequency square-wave injection, introduces a harmonic current extraction technique based on multiple synchronous reference frame transformations to separate the fifth and seventh harmonic components accurately; then, according to the established harmonic voltage compensation equation, generates targeted compensation voltage commands; finally, further precisely suppresses the corresponding harmonic currents through a sixth-order QPR controller connected in parallel with the current proportional-integral (PI) controller. This paper comprehensively establishes the mathematical models for harmonic extraction and voltage compensation, and conducts a detailed analysis of the parameter design of the sixth-order QPR controller. Simulation results demonstrate that the proposed strategy can significantly suppress stator current distortion, effectively reduce torque and speed ripples, and substantially improve rotor position estimation accuracy, thereby verifying the superiority of the novel harmonic-suppression-based sensorless control strategy.

## 1. Introduction

In recent years, interior permanent magnet synchronous motors (IPMSM) have garnered significant attention from researchers due to their high efficiency, high power factor, and excellent dynamic performance [1,2]. To reduce costs and enhance system fault tolerance, sensorless control has become a research focus and has been applied in fields such as electric vehicles and air-conditioning compressors [3,4].

Sensorless control techniques for IPMSM are primarily categorized into zero-to-low speed and medium-to-high speed regimes [5]. In the zero-to-low speed regime, the low signal-to-noise ratio of back electromotive force (EMF) necessitates the injection of high-frequency (HF) voltage signals to induce HF currents containing rotor position information. The rotor position angle is subsequently extracted through position demodulation for feedback. Based on the type of injected signal, these techniques can be divided into rotating sinusoidal HF injection [6,7,8,9], pulsating sinusoidal HF injection [10,11,12], and pulsating square-wave HF injection [13,14,15,16,17].

Rotating sinusoidal and pulsating sinusoidal HF injection methods inject HF voltage signals into the stationary or rotating reference frame. Typically, a high-pass filter (HPF) is used to extract the HF induced current, followed by a low-pass filter (LPF) to mitigate the impact of residual HF components on the extracted position information. However, the use of filters introduces phase delays, which degrade the performance of the current control loop [18]. In contrast, the pulsating square-wave HF injection method allows the injection frequency to be set to one-quarter of the sampling frequency or as high as the pulse width modulation (PWM) carrier frequency, eliminating the need for HPF and LPF.

In the pulsating square-wave injection strategy, high injection frequencies, inverter nonlinearities, slot effects, and magnetic saturation can lead to current harmonics, which affect the stability and estimation accuracy of the strategy. Therefore, suppressing stator current harmonics is essential. Reference [19] provides a detailed analysis of the impact of inverter nonlinearities on injected voltage, induced current, and position estimation errors, proposing a method to mitigate these effects by adjusting the HF current ripple. In the medium-to-high speed regime, the back-EMF signal containing rotor position information in sliding mode observers is affected by direct current (DC)-link voltage fluctuations, introducing harmonics that distort rotor position estimation. In [20], from the perspective that the back electromotive force signal containing rotor position information in the sliding mode observer for the medium-high speed region is affected by voltage fluctuations of the DC-link, thereby introducing harmonics that cause distortion in rotor position error, the harmonics introduced by the DC-link are effectively suppressed by using a complex band-pass filter instead of a traditional LPF. Similarly, Reference [21] uses a BRLS adaptive filter to suppress the fifth and seventh voltage harmonics in the back-EMF. Reference [22] proposes a voltage harmonic suppression strategy based on adaptive synchronous rotating frame transformation, injecting voltage compensation into the driver to suppress current harmonics in the corresponding reference frame. While these methods achieve some success in suppressing current harmonics, they face two main issues: one is their applicability primarily to sensorless methods based on back-EMF for rotor position estimation, and the other is the failure to account for additional alternating current (AC) harmonic components introduced by the fundamental wave in harmonic voltage compensation.

To reduce high-order stator current harmonics, this paper proposes a harmonic extraction and voltage compensation strategy for IPMSM with pulsating square-wave high-frequency injection, based on sixth-order QPR control. By transforming the rotating coordinate system to the fifth and seventh rotating reference frames, the corresponding harmonic currents are extracted using a low-pass filter. Harmonic voltages are then compensated, and a proportional resonant controller is employed to suppress the additional harmonics introduced.

## 2. Rotor Position Extraction Based on High-Frequency Square-Wave Voltage Injection

### 2.1. Square-Wave Injection Position Estimation Without Filter

The voltage mathematical model of an interior permanent magnet synchronous motor in the rotating coordinate system is

(1) $$\left[\begin{array}{c} u_{d}\\ u_{q} \end{array}\right]=\left[\begin{array}{cc} R_{s}+pL_{d} & -\omega_{e}L_{q}\\ \omega_{e}L_{d} & R_{s}+pL_{q} \end{array}\right]\left[\begin{array}{c} i_{d}\\ i_{q} \end{array}\right]+\left[\begin{array}{c} 0\\ \omega_{e}\psi_{f} \end{array}\right]$$

where p is the differential operator, $L_{d}$ and $L_{q}$ are the stator inductances, $i_{d}$ and $i_{q}$ are the stator currents and voltages, $R_{s}$ is the stator resistance, $\omega_{e}$ is the electrical angular velocity, $\psi_{f}$ is the permanent magnet flux linkage.

The traditional high-frequency square-wave voltage signal injection strategy injects a square-wave voltage proportional to the PWM period into the d-axis of the rotating coordinate system to excite the HF response current containing rotor position information, and obtains effective rotor position information through subsequent signal processing and position observer steps.

The high-frequency square-wave signal with amplitude $U_{h\_in}$ injected into the d-axis of the estimated coordinate system can be expressed as

(2) $$\left[\begin{array}{c} u_{d\_h}^{\hat{j}}\\ u_{q\_h}^{\hat{j}} \end{array}\right]=\left[\begin{array}{c} {\left(-1\right)}^{n}U_{h\_in}\\ 0 \end{array}\right]$$

where $u_{d\_h}^{\hat{j}}$ and $u_{q\_h}^{\hat{j}}$ represent the HF voltage components injected in the estimated rotating coordinate system, the superscript $\hat{j}$ and subscript h represent the estimated coordinate system and HF injection quantity, n represents the sampling number.

When the motor operates in the zero-low-speed range, the fundamental frequency component generated by the permanent magnet flux linkage is much smaller than the injected HF component. Thus, the terms related to the stator impedance and fundamental frequency can be ignored, and Equation (1) can be simplified as

(3) $$p\left[\begin{array}{c} i_{d\_h}\\ i_{q\_h} \end{array}\right]=\left[\begin{array}{cc} \frac{1}{L_{d}} & 0\\ 0 & \frac{1}{L_{q}} \end{array}\right]\left[\begin{array}{c} u_{d\_h}\\ u_{q\_h} \end{array}\right]$$

where $i_{d\_h}$, $i_{q\_h}$, $u_{d\_h}$, and $u_{q\_h}$ are the HF current and voltage components in the dq-axis.

The extraction of rotor position requires obtaining the HF response current in the stationary axis system, which can be obtained through rotor position coordinate transformation

(4) $$p\left[\begin{array}{c} i_{\alpha \_h}\\ i_{\beta \_h} \end{array}\right]=T(\theta_{e})\left[\begin{array}{cc} \frac{1}{L_{d}} & 0\\ 0 & \frac{1}{L_{q}} \end{array}\right]T^{-1}({\tilde{\theta }}_{e})\left[\begin{array}{c} u_{d\_h}^{\hat{j}}\\ u_{q\_h}^{\hat{j}} \end{array}\right]$$

where $i_{\alpha \_h}$ and $i_{\beta \_h}$ are the HF currents in the αβ-axis system, T is the coordinate transformation matrix, ${\tilde{\theta }}_{e}={\hat{\theta }}_{e}-\theta_{e}$ is the estimated rotor position error.

By further deriving from Equation (4), the differential of the HF response current in the αβ-axis system, after simplification, yields the HF current variation over two consecutive periods

(5) $$\left[\begin{array}{c} \Delta i_{\alpha \_h}\\ \Delta i_{\beta \_h} \end{array}\right]=\frac{u_{d\_h}^{\hat{j}}T_{h}}{L_{d}L_{q}}\left[\begin{array}{c} \sum L\cos{\hat{\theta }}_{e}-\Delta L\cos(\theta_{e}+{\tilde{\theta }}_{e})\\ \sum L\sin({\hat{\theta }}_{e})-\Delta L\sin(\theta_{e}+{\tilde{\theta }}_{e}) \end{array}\right]$$

where $T_{h}$ is the injected HF square-wave signal period, $\sum L=(L_{d}+L_{q})/2$ is the average inductance, $\Delta L=(L_{d}-L_{q})/2$ is the half-difference inductance.

### 2.2. Rotor Position Signal Extraction

The envelope of the HF current variation in the stationary αβ-axis is extracted using the sign function of the injected square-wave voltage as follows

(6) $$\left[\begin{array}{c} i_{\alpha \_h}^{c}\\ i_{\beta \_h}^{c} \end{array}\right]=\frac{sign(u_{d\_h}^{\hat{j}})T_{h}u_{d\_h}^{\hat{j}}}{L_{d}L_{q}}\left[\begin{array}{c} \sum L\cos{\hat{\theta }}_{e}-\Delta L\cos(\theta_{e}+{\tilde{\theta }}_{e})\\ \sum L\sin{\hat{\theta }}_{e}-\Delta L\sin(\theta_{e}+{\tilde{\theta }}_{e}) \end{array}\right]$$

**Figure 1:**
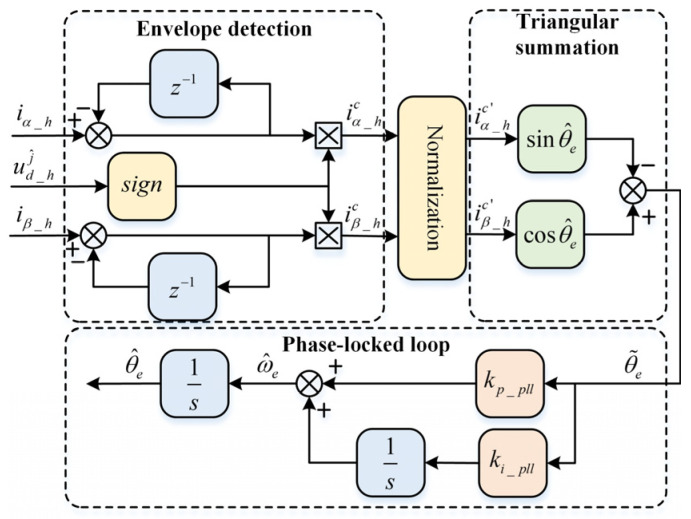
Decoupling of position error signal in delta operation.

The HF current in the estimated αβ-axis is normalized to process the two envelope functions, as illustrated in Figure 1. Subsequently, the phase-locked loop method of summing sine and cosine functions is used to demodulate the position error signal, then

(7) $$i_{\alpha \_h}^{c^{\prime }}\sin{\hat{\theta }}_{e}-i_{\beta \_h}^{c^{\prime }}\cos{\hat{\theta }}_{e}=\sin({\hat{\theta }}_{e}-\theta_{e})\approx {\tilde{\theta }}_{e}$$

where $i_{\alpha \_h}^{c^{\prime }}$ and $i_{\beta \_h}^{c^{\prime }}$ are the normalized envelope functions.

Therefore, the transfer function of the proportional-integral (PI) phase-locked loop structure as the position observer can be expressed as

(8) $$\frac{{\hat{\theta }}_{e}}{\theta_{e}}=\frac{k_{p\_pll}s+k_{i\_pll}}{s^{2}+k_{p\_pll}s+k_{i\_pll}}$$

where $k_{p\_pll}=2\xi \omega_{n}$, $k_{i\_pll}=\omega_{n}^{2}$, $\omega_{n}$ is the desired bandwidth of the phase-locked loop, ξ is the damping ratio.

## 3. Analysis of Predictive Current Harmonic Suppression Based on Multiple Coordinate Systems

### 3.1. Stator Current Harmonic Analysis

Typically, the IPMSM stator employs a three-phase winding configuration, distributed with a 120° phase difference. It does not contain 3rd, 9th, 15th, etc., or even-order stator current harmonics. Due to the nonlinear characteristics of the inverter, motor slot effects, and other factors, a large number of low-order current harmonics are generated, causing current distortion and thereby increasing torque and speed pulsations. Among them, the 5th and 7th stator current harmonics are dominant. Thus, the three-phase winding stator currents can be expressed as

(9) $$\left\{\begin{array}{l} \begin{array}{l} i_{a}=I_{1\mathrm{th}}\cos(\omega_{e}t+\theta_{1})+I_{5\mathrm{th}}\cos(\omega_{5th}t+\theta_{5})\\ \;+I_{7\mathrm{th}}\cos(7\omega_{7th}t+\theta_{7}) \end{array}\\ \begin{array}{l} i_{b}=I_{1\mathrm{th}}\cos(\omega_{e}t+\theta_{1}-\frac{2\pi }{3})+I_{5\mathrm{th}}\cos(\omega_{5th}t+\theta_{5}+\frac{2\pi }{3})\\ \;+I_{7\mathrm{th}}\cos(\omega_{7th}t+\theta_{7}-\frac{2\pi }{3}) \end{array}\\ \begin{array}{l} i_{c}=I_{1\mathrm{th}}\cos(\omega_{e}t+\theta_{1}+\frac{2\pi }{3})+I_{5\mathrm{th}}\cos(\omega_{5th}t+\theta_{5}-\frac{2\pi }{3})\\ \;+I_{7\mathrm{th}}\cos(\omega_{7th}t+\theta_{7}+\frac{2\pi }{3}) \end{array} \end{array}\right.$$

where $I_{1\mathrm{th}}$, $I_{5\mathrm{th}}$, and $I_{7\mathrm{th}}$ are the amplitudes of the fundamental, 5th, and 7th current harmonics; $\theta_{1}$, $\theta_{5}$, and $\theta_{7}$ are the initial phases of the fundamental, 5th, and 7th current harmonics; and $\omega_{5th}=5\omega_{e}$ and $\omega_{7th}=7\omega_{e}$ are the angular frequencies of the 5th and 7th harmonics, respectively.

Since the injected HF voltage signal introduces an HF current component into the fundamental current waveform, and considering the periodicity of the square-wave injection along with its frequency being significantly higher than the fundamental frequency, the fundamental component can be approximated algebraically. Accordingly, the fundamental signal containing harmonics is processed by

(10) $${\hat{i}}_{dql}(k+1)=\frac{{\hat{i}}_{dq}(k+1)+{\hat{i}}_{dq}(k)}{2}$$

Thus, the current equation in the estimated dq-axis containing the 5th and 7th stator current harmonics is obtained

(11) $$\left\{\begin{array}{c} \begin{array}{l} {\hat{i}}_{dl}=I_{1\mathrm{th}}\cos\theta_{1}+I_{5\mathrm{th}}\cos(-6{\hat{\omega }}_{e}t+\theta_{5})\\ \;+I_{7\mathrm{th}}\cos(6{\hat{\omega }}_{e}t+\theta_{7}) \end{array}\\ \begin{array}{l} {\hat{i}}_{ql}=I_{1\mathrm{th}}\sin\theta_{1}+I_{5\mathrm{th}}\sin(-6{\hat{\omega }}_{e}t+\theta_{5})\\ \;+I_{7\mathrm{th}}\sin(6{\hat{\omega }}_{e}t+\theta_{7}) \end{array} \end{array}\right.$$

After transformation to the estimated dq-axis, the angular frequencies of the 5th and 7th stator current harmonics become $-6{\hat{\omega }}_{e}$ and $6{\hat{\omega }}_{e}$, respectively.

From Equation (11) combined with Equation (1), the estimated dq-axis voltage error model containing the 5th and 7th stator current harmonics is obtained

(12) $$\left\{\begin{array}{c} \begin{array}{l} \Delta u_{dl}=(6{\hat{\omega }}_{e}L_{d}-{\hat{\omega }}_{e}L_{q})I_{5\mathrm{th}}\sin(-6{\hat{\omega }}_{e}t+\theta_{5})\\ \;\;+R_{s}I_{5\mathrm{th}}\cos(-6{\hat{\omega }}_{e}t+\theta_{5})\\ \;\;-(6{\hat{\omega }}_{e}L_{d}+{\hat{\omega }}_{e}L_{q})I_{7\mathrm{th}}\sin(6{\hat{\omega }}_{e}t+\theta_{7})\\ \;\;+R_{s}I_{7\mathrm{th}}\cos(6{\hat{\omega }}_{e}t+\theta_{7}) \end{array}\\ \begin{array}{l} \Delta u_{ql}=({\hat{\omega }}_{e}L_{q}-6{\hat{\omega }}_{e}L_{d})I_{5\mathrm{th}}\cos(-6{\hat{\omega }}_{e}t+\theta_{5})\\ \;\;+R_{s}I_{5\mathrm{th}}\sin(-6{\hat{\omega }}_{e}t+\theta_{5})\\ \;\;+(6{\hat{\omega }}_{e}L_{q}+{\hat{\omega }}_{e}L_{d})I_{7\mathrm{th}}\cos(6{\hat{\omega }}_{e}t+\theta_{7})\\ \;\;+R_{s}I_{7\mathrm{th}}\sin(6{\hat{\omega }}_{e}t+\theta_{7}) \end{array} \end{array}\right.$$

where $\Delta u_{dl}$ and $\Delta u_{ql}$ are the error voltages in the estimated dq-axis system with the fundamental current component removed.

### 3.2. Current Harmonic Extraction

**Figure 2:**
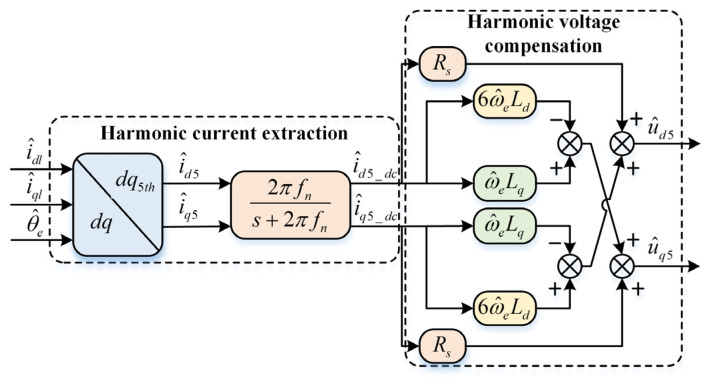
Block diagram of harmonic current extraction and voltage compensation section.

**Figure 3:**
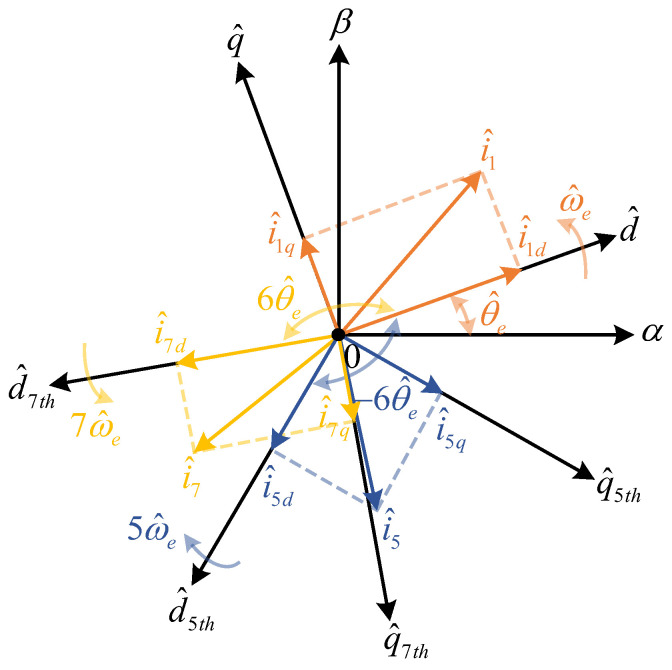
Schematic of the estimated rotational coordinate system for 5th and 7th harmonics.

The current harmonic extraction strategy is shown in Figure 2. To convert the harmonic AC components in the estimated dq-axis to DC components for extraction and subsequent voltage compensation, the currents in the estimated dq-axis are transformed to the 5th and 7th dq-axis systems, respectively. The relationships between different order rotating coordinate systems are shown in Figure 3. The transformation matrices for the 5th and 7th axis systems are as follows

(13) $$T_{dq\to dq5th}=\left[\begin{array}{cc} \cos6{\hat{\omega }}_{e}t & -\sin6{\hat{\omega }}_{e}t\\ \sin6{\hat{\omega }}_{e}t & \cos6{\hat{\omega }}_{e}t \end{array}\right]$$

(14) $$T_{dq\to dq7th}=\left[\begin{array}{cc} \cos6{\hat{\omega }}_{e}ct & \sin6{\hat{\omega }}_{e}t\\ -\sin6{\hat{\omega }}_{e}t & \cos6{\hat{\omega }}_{e}t \end{array}\right]$$

where $T_{dq\to dq5th}$ and $T_{dq\to dq7th}$ are the coordinate transformation matrices, respectively.

By transforming through Equations (13) and (14) to the 5th and 7th dq-axis systems, the following harmonic currents can be obtained

(15) $$\left\{\begin{array}{c} \begin{array}{l} {\hat{i}}_{d5}=I_{1\mathrm{th}}\cos(6{\hat{\omega }}_{e}t+\theta_{1})+I_{7\mathrm{th}}\cos(12{\hat{\omega }}_{e}t+\theta_{7})\\ \;\;+I_{5\mathrm{th}}\cos\theta_{5} \end{array}\\ \begin{array}{l} {\hat{i}}_{q5}=I_{1\mathrm{th}}\sin(6{\hat{\omega }}_{e}t+\theta_{1})+I_{7\mathrm{th}}\sin(12{\hat{\omega }}_{e}t+\theta_{7})\\ \;\;+I_{5\mathrm{th}}\sin\theta_{5} \end{array} \end{array}\right.$$

(16) $$\left\{\begin{array}{l} \begin{array}{l} {\hat{i}}_{d7}=I_{1\mathrm{th}}\cos(6{\hat{\omega }}_{e}t-\theta_{1})+I_{5\mathrm{th}}\cos(12{\hat{\omega }}_{e}t+\theta_{5})\\ \;\;+I_{7\mathrm{th}}\cos\theta_{7} \end{array}\\ \begin{array}{l} {\hat{i}}_{q7}=-I_{1\mathrm{th}}\sin(6{\hat{\omega }}_{e}t-\theta_{1})-I_{5\mathrm{th}}\sin(12{\hat{\omega }}_{e}t+\theta_{5})\\ \;\;+I_{7\mathrm{th}}\sin\theta_{7} \end{array} \end{array}\right.$$

where ${\hat{i}}_{d5}$ and ${\hat{i}}_{q5}$ are the d-and q-axis currents in the 5th dq-axis system, and ${\hat{i}}_{d7}$ and ${\hat{i}}_{q7}$ are the d-and q-axis currents in the 7th dq-axis system.

After coordinate transformation, the AC components corresponding to the 5th and 7th harmonics are converted to DC components in the corresponding 5th and 7th dq-axis systems, while the fundamental and non-corresponding order harmonics become AC components with corresponding angular velocities of $6{\hat{\omega }}_{e}$ and $12{\hat{\omega }}_{e}$, respectively. An LPF is used to filter out the HF AC components, obtaining the harmonic DC components in the corresponding axis systems for subsequent voltage transformation and harmonic compensation operations.

The corresponding currents in the 5th and 7th dq-axis systems after low-pass filtering are as follows

(17) $$\left\{\begin{array}{c} {\hat{i}}_{d5\_dc}=I_{5\mathrm{th}}\cos\theta_{5}\\ {\hat{i}}_{q5\_dc}=I_{5\mathrm{th}}\sin\theta_{5} \end{array}\right.$$

(18) $$\left\{\begin{array}{c} {\hat{i}}_{d7\_dc}=I_{7\mathrm{th}}\cos\theta_{7}\\ {\hat{i}}_{q7\_dc}=I_{7\mathrm{th}}\sin\theta_{7} \end{array}\right.$$

where ${\hat{i}}_{d5\_dc}$ and ${\hat{i}}_{q5\_dc}$ are the DC components in the 5th dq-axis system, and ${\hat{i}}_{d7\_dc}$ and ${\hat{i}}_{q7\_dc}$ are the DC components in the 7th dq-axis system.

### 3.3. Voltage Compensation Based on Multi-Coordinate System Current Harmonic Extraction

To compensate for the extracted harmonic currents of the two orders, the currents in the estimated dq-axis system are transformed to the 5th and 7th dq-axis systems. To simplify the analysis, only the 5th harmonic voltage compensation is taken as an example below; the 7th harmonic compensation follows similarly. The error voltage equation in the 5th dq-axis system can be obtained by processing the voltage error equation in the estimated dq-axis system, as follows

(19) $$\left\{\begin{array}{c} \begin{array}{l} \Delta u_{d5}=R_{s}[I_{5th}\cos\theta_{5}+I_{7th}\cos(12{\hat{\omega }}_{e}t+\theta_{7})]\\ \;\;-{\hat{\omega }}_{e}L_{q}[I_{5th}\sin\theta_{5}+I_{7th}\sin(12{\hat{\omega }}_{e}t+\theta_{7})]\\ \;\;+6{\hat{\omega }}_{e}L_{q}[I_{5th}\sin\theta_{5}-I_{7th}\sin(12{\hat{\omega }}_{e}t+\theta_{7})] \end{array}\\ \begin{array}{l} \Delta u_{q5}=R_{s}[I_{5th}\sin\theta_{5}+I_{7th}\sin(12{\hat{\omega }}_{e}t+\theta_{7})]\\ \;\;+{\hat{\omega }}_{e}L_{q}[I_{5th}\cos\theta_{5}+I_{7th}\cos(12{\hat{\omega }}_{e}t+\theta_{7})]\\ \;\;+6{\hat{\omega }}_{e}L_{q}[I_{5th}\cos\theta_{5}-I_{7th}\cos(12{\hat{\omega }}_{e}t+\theta_{7})] \end{array} \end{array}\right.$$

where $\Delta u_{d5}$ and $\Delta u_{q5}$ are the error voltages in the 5th dq-axis system.

According to Equation (19), by eliminating the $I_{7th}\cos(12{\hat{\omega }}_{e}t+\theta_{7})$ and $I_{7th}\sin(12{\hat{\omega }}_{e}t+\theta_{7})$ terms of the 5th order error harmonic voltage, the harmonic voltage compensation equation can be constructed as

(20) $$\left\{\begin{array}{c} {\hat{u}}_{d5}=R_{s}{\hat{i}}_{d5\_dc}-{\hat{\omega }}_{e}L_{q}{\hat{i}}_{q5\_dc}+6{\hat{\omega }}_{e}L_{d}{\hat{i}}_{q5\_dc}\\ {\hat{u}}_{q5}=R_{s}{\hat{i}}_{q5\_dc}+{\hat{\omega }}_{e}L_{q}{\hat{i}}_{d5\_dc}-6{\hat{\omega }}_{e}L_{d}{\hat{i}}_{d5\_dc} \end{array}\right.$$

The voltage compensation section block diagram is shown in Figure 2. The estimated dq-axis currents with the HF response current removed are transformed to the corresponding order dq-axis systems via multi-coordinate system transformation matrix. These currents are then filtered through an LPF to eliminate other order AC HF components while retaining the corresponding order DC components. The extracted harmonic DC components are converted to voltages using the constructed harmonic voltage compensation strategy. Finally, they are inversely transformed back to the estimated dq-axis system via multi-coordinate system inverse transformation, and then dq-axis voltage compensation is performed.

## 4. Multi-Coordinate-System Predictive Current Harmonic Voltage Compensation Strategy Based on Sixth-Order Quasi-Proportional Resonant Control

### 4.1. Current Loop Control Based on Sixth-Order Quasi-Proportional Resonant Control

The fifth-order stator current harmonic is taken as an example to analyze the harmonic suppression mechanism during the processes of harmonic current extraction and voltage compensation. During the extraction of fifth- and seventh-order current harmonics, a first-order LPF is typically employed. To ensure a broad speed regulation range and mitigate the delay introduced by the LPF, its cutoff frequency is not set excessively low. As the fundamental component $I_{1\mathrm{th}}\cos(6{\hat{\omega }}_{e}t+\theta_{1})$ constitutes a significantly larger proportion than other AC harmonics and operates at a lower frequency than the 7th harmonic component $I_{7\mathrm{th}}\cos(12{\hat{\omega }}_{e}t+\theta_{7})$, the extracted current harmonics containing the fundamental AC component can be expressed as

(21) $$\left\{\begin{array}{c} {\hat{i}}_{d5\_dch}=I_{5\mathrm{th}}\cos\theta_{5}+I_{1\mathrm{th}}\cos(6{\hat{\omega }}_{e}t+\theta_{1})\\ {\hat{i}}_{q5\_dch}=I_{5\mathrm{th}}\sin\theta_{5}+I_{1\mathrm{th}}\sin(6{\hat{\omega }}_{e}t+\theta_{1}) \end{array}\right.$$

Equation (21) reveals that the AC components introduced into the fifth-order dq-axis are $I_{1\mathrm{th}}\cos(6{\hat{\omega }}_{e}t+\theta_{1})$ and $I_{1\mathrm{th}}\sin(6{\hat{\omega }}_{e}t+\theta_{1})$. By analysing these two terms alone, the voltage fluctuations introduced in the subsequent voltage compensation strategy are shown in Equation (22)

(22) $$\left\{\begin{array}{c} \begin{array}{l} \Delta {\hat{u}}_{d5\_h}=R_{s}I_{1\mathrm{th}}\cos(6{\hat{\omega }}_{e}t+\theta_{1})-{\hat{\omega }}_{e}L_{q}I_{1\mathrm{th}}\sin(6{\hat{\omega }}_{e}t+\theta_{1})\\ +6{\hat{\omega }}_{e}L_{d}I_{1\mathrm{th}}\sin(6{\hat{\omega }}_{e}t+\theta_{1}) \end{array}\\ \begin{array}{l} \Delta {\hat{u}}_{q5\_h}=R_{s}I_{1\mathrm{th}}\sin(6{\hat{\omega }}_{e}t+\theta_{1})+{\hat{\omega }}_{e}L_{q}I_{1\mathrm{th}}\cos(6{\hat{\omega }}_{e}t+\theta_{1})\\ -6{\hat{\omega }}_{e}L_{d}I_{1\mathrm{th}}\cos(6{\hat{\omega }}_{e}t+\theta_{1}) \end{array} \end{array}\right.$$

**Figure 4:**
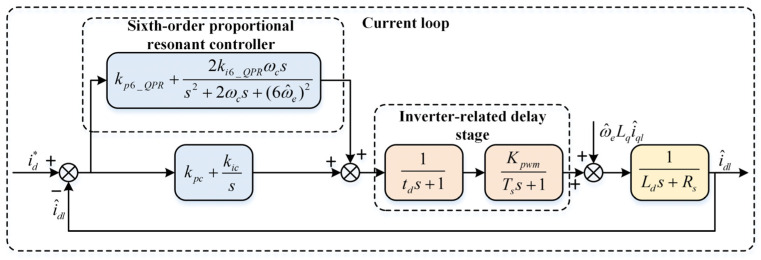
Transfer function of the current loop based on 6th QPR control.

It can be observed that the voltage fluctuations induced by the fundamental component contain a sixth-order current harmonic with angular velocity $6{\hat{\omega }}_{e}$. Subsequently, converting $\Delta {\hat{u}}_{d5\_h}$ and $\Delta {\hat{u}}_{q5\_h}$ to the estimated dq-axis system reveals that the sixth-order current harmonic 6n(n=1,2,3…) persists even after voltage compensation, manifesting as a seventh-order stator current harmonic 6n±1. To further suppress the 5th and 7th stator current harmonics, a control scheme employing a QPR controller in parallel with the current loop PI controller was adopted. The QPR controller suppresses the 5th and 7th stator current harmonics, while the PI controller continues to serve as the closed-loop control for the current inner loop. The resonance frequency of the QPR controller is set to $6{\hat{\omega }}_{e}$.The block diagram of the current loop transfer function based on the sixth-order QPR controller is shown in Figure 4.

Based on the current loop transfer function block diagram, setting $K_{PWM}$ to 1 and merging the delay section with the inverter section, the open-loop transfer function of the current loop is expressed as follows

(23) $$G(s)=[G_{PI}(s)+G_{6QPR}(s)]\frac{1}{\left(1.5T_{s}s+1)(L_{d}s+R_{s}\right)}$$

where $G_{6QPR}(s)$ represents the transfer function of the 6th-order QPR controller, $K_{PWM}$ denotes the inverter gain, and $T_{s}$ denotes the open-loop period. Here, $t_{d}=T_{s}$ denotes the delay introduced by sampling and computation.

### 4.2. Parameter Tuning Analysis of the Quasi-Proportional Resonant Controller

The current-loop transfer function block diagram in Figure 4 clearly demonstrates that the design of the sixth-order QPR controller primarily depends on $k_{p6\_QPR}$, $k_{i6\_QPR}$, and $\omega_{c}$. Consequently, parameter design for the proportional resonance controller is particularly crucial. By analysing the Bode plot through variable control methods, the values for the sixth-order QPR controller parameters in this study were determined. Given a speed of 200r/min then ${\hat{\omega }}_{e}=(80\pi /3)$ rad/s.

**Figure 5:**
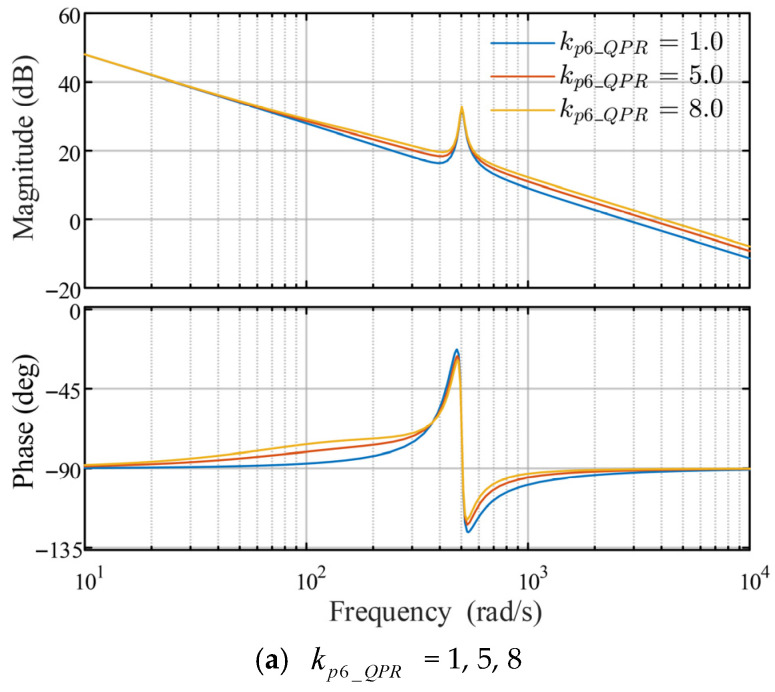

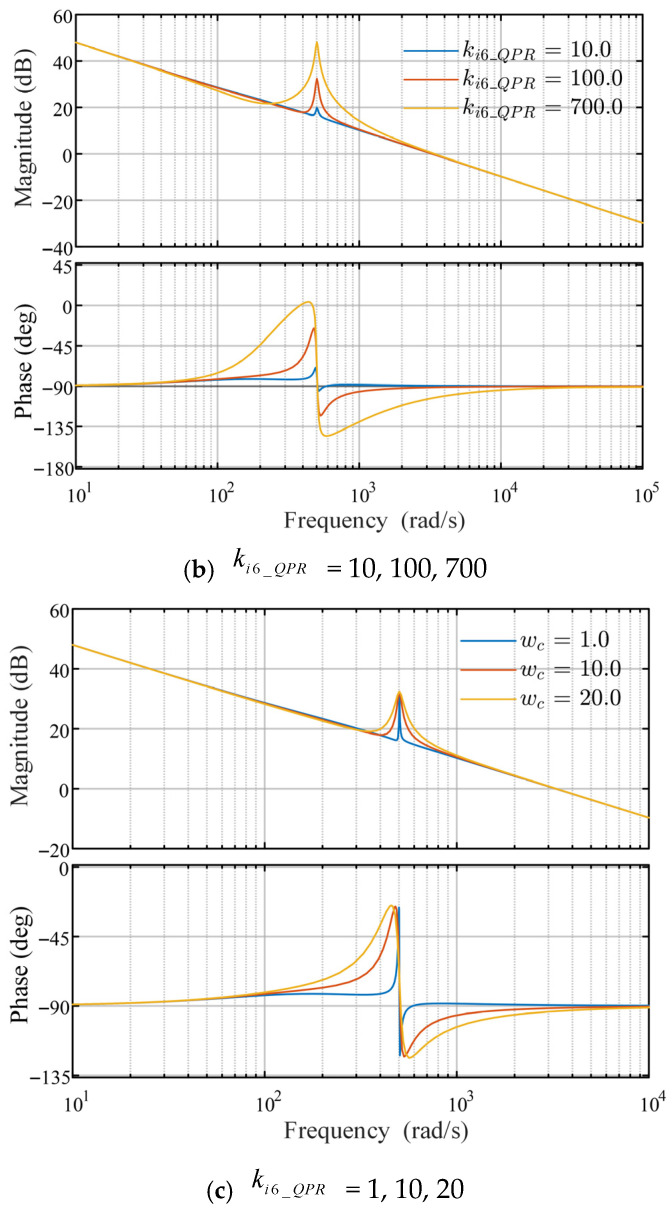
Bode plot of the current open-loop transfer function based on 6th QPR under different parameters.

When the parameter $k_{p6\_QPR}$ is set to 1, 5, and 8, respectively, as shown in Figure 5a, the resonance peak at the resonant frequency $6{\hat{\omega }}_{e}$ is increased, thereby effectively suppressing the 6th-order current harmonic. Consequently, the parameter $k_{p6\_QPR}$ cannot be eliminated. However, increasing its value does not significantly enhance harmonic suppression, so $k_{p6\_QPR}$ should not be set excessively high.

As shown in Figure 5b,c, the corresponding Bode plots for parameters $k_{i6\_QPR}$ and $\omega_{c}$ are depicted. When the value of parameter $k_{i6\_QPR}$ increases, the resonance peak at the frequency suppression point rises significantly, thereby reducing steady-state error. Parameter $\omega_{c}$ primarily influences the bandwidth at the resonance frequency; if too small, it cannot adequately suppress harmonics under varying rotational speeds. After comprehensive consideration, the values selected for this study are: $k_{p6\_QPR}$ = 5, $k_{i6\_QPR}$ = 400, and $\omega_{c}$ = 15.

## 5. Simulation Results and Analysis

**Figure 6:**
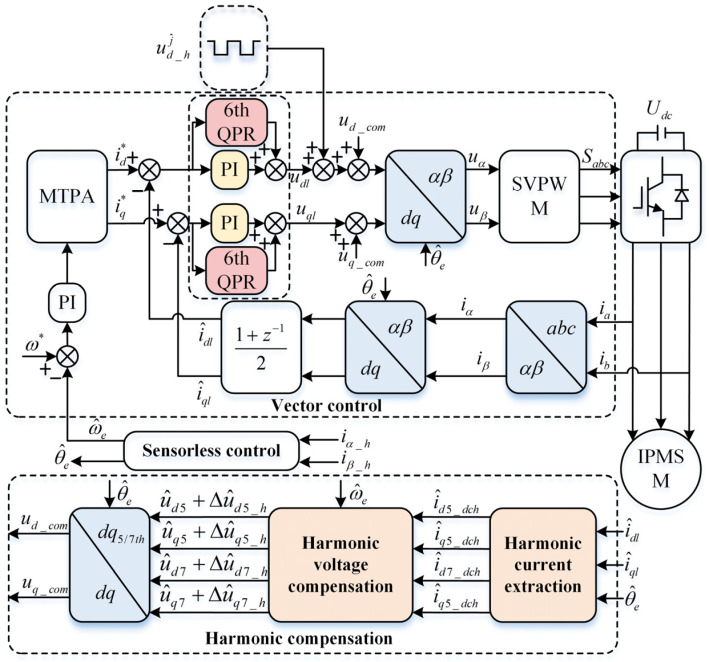
Block diagram of the proposed harmonic suppression strategy.

The block diagram of the proposed sensorless control strategy for harmonic current extraction and voltage compensation based on the sixth-order QPR controller is shown in Figure 6. To validate the effectiveness of the proposed harmonic suppression strategy for square-wave injection sensorless control, as well as its improvement in torque and speed fluctuation, simulation experiments were conducted in MATLAB/Simulink. The parameters of the IPMSM are listed in Table 1. The simulation adopts the maximum torque per ampere (MTPA) control method to effectively improve the inverter’s efficiency. The dead-time is set to 5 μs, the injected signal frequency is 5 kHz, and the amplitude is 40 V.

**Figure 7:**
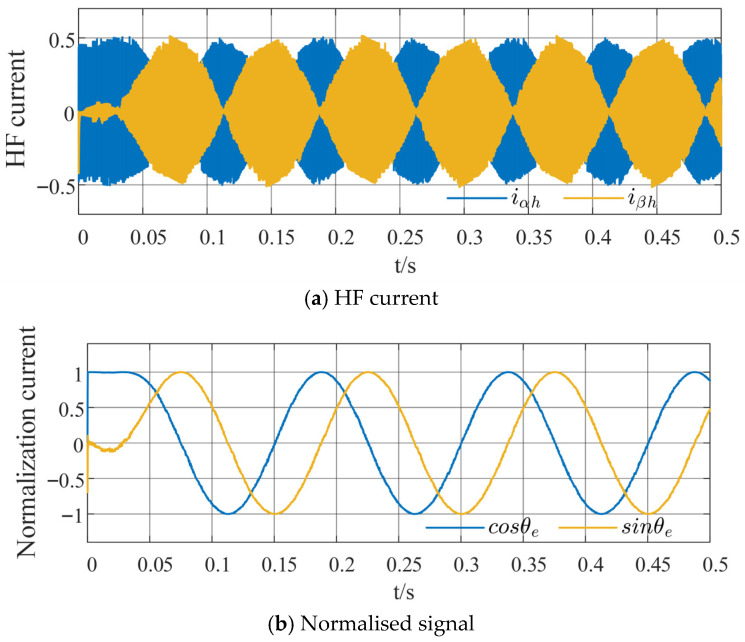
Proposed harmonic suppression strategy.

Figure 7 illustrates the HF response current waveform and the normalized orthogonal signal extracted under the proposed harmonic suppression strategy based on the sixth-order QPR controller for HFSWI in a permanent magnet synchronous motor (PMSM) sensorless control, at a given speed (100 r/min) and load (5 N·m) condition. The HF response current containing rotor position information is extracted directly, without requiring an HPF, as shown in Figure 7a. Additionally, the normalized orthogonal signal in Figure 7b is relatively smooth, facilitating subsequent signal processing.

**Figure 8:**
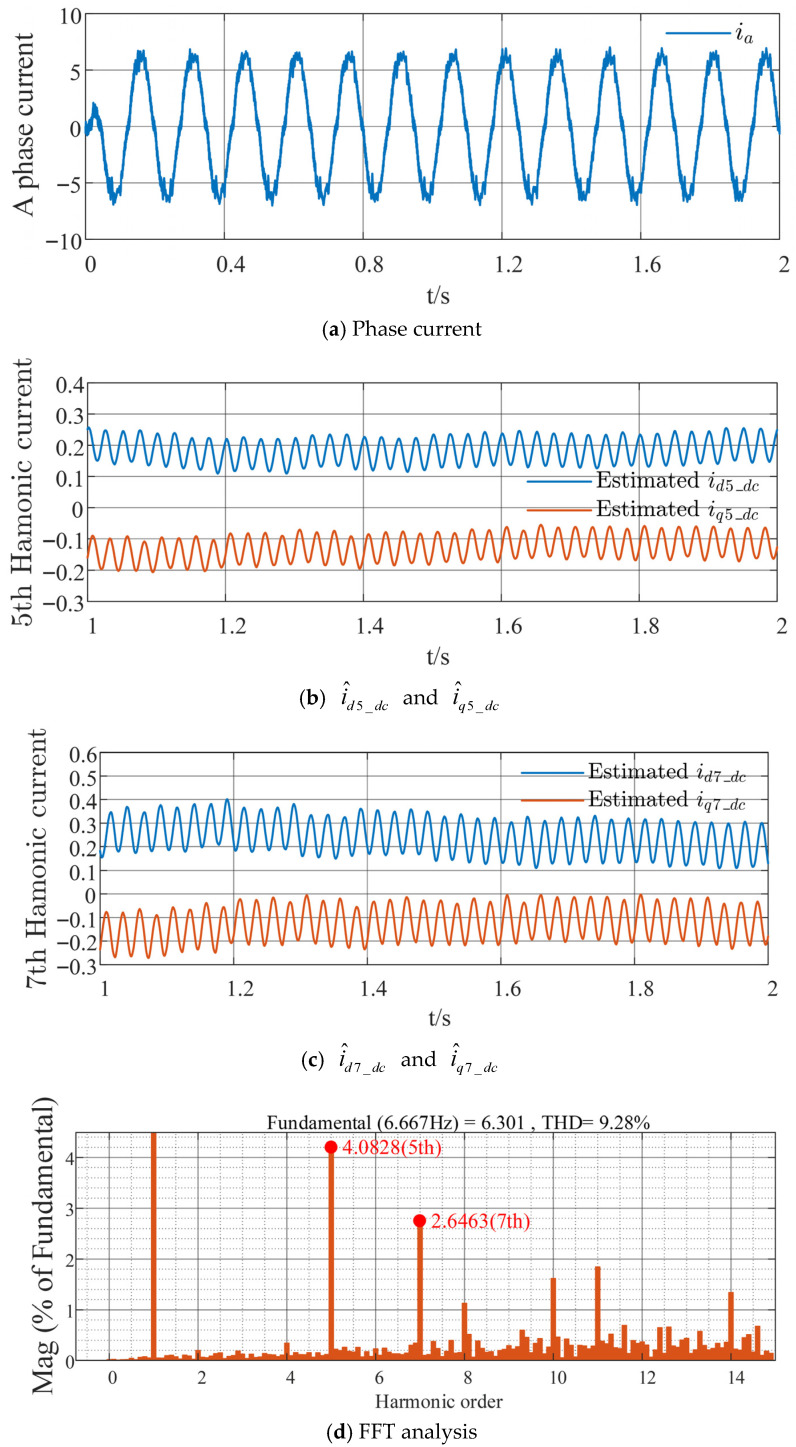
Experimental waveforms of the traditional HFSWI strategy at a given speed of 100 r/min and load torque of 7 N·m.

**Figure 9:**
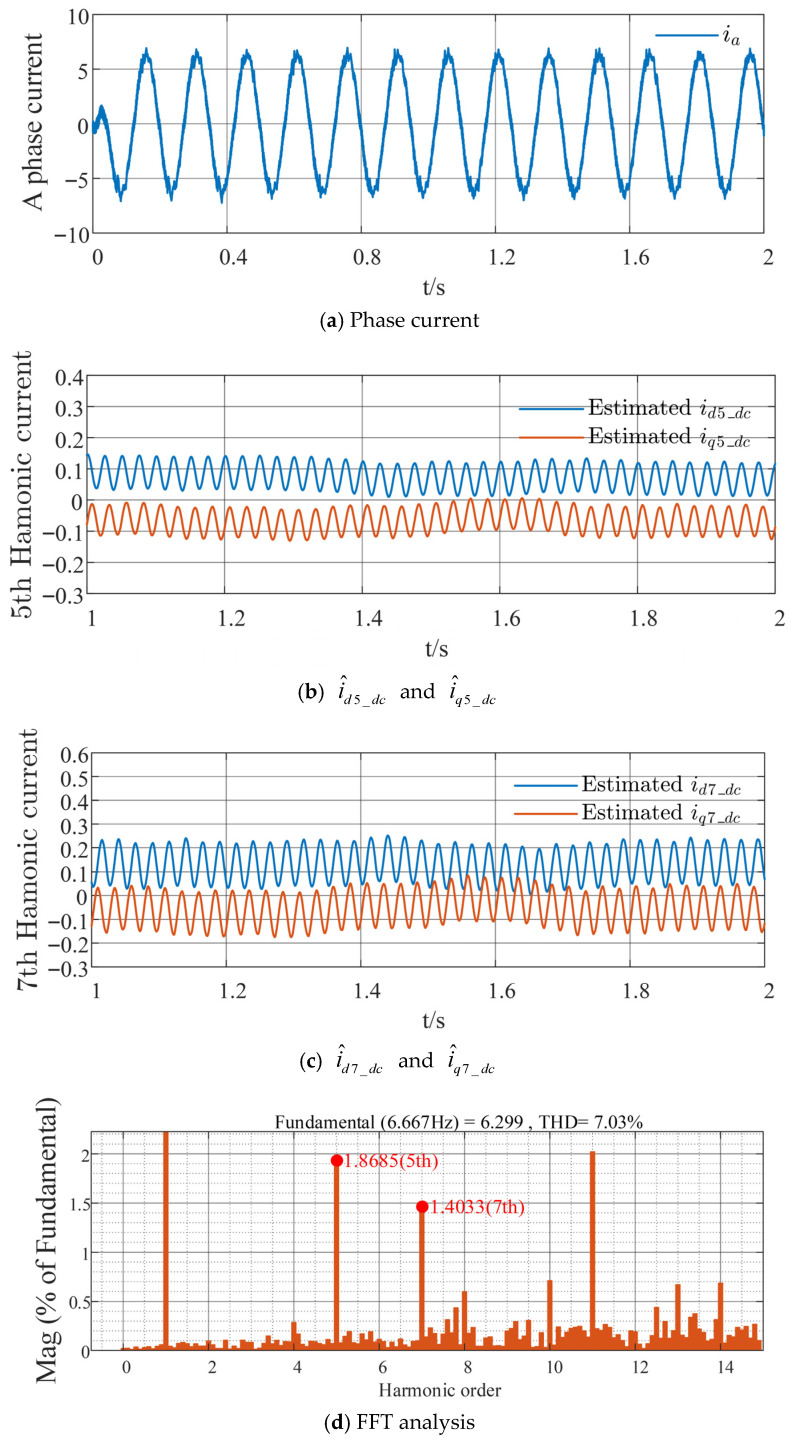
Experimental waveforms of the proposed strategy at a given speed of 100 r/min and load torque of 7 N·m.

The traditional HFSWI strategy is compared with the proposed harmonic suppression method based on the sixth-order QPR controller, as shown in Figure 8 and Figure 9, respectively. As shown in Figure 8a and Figure 9a, the phase current distortion with the proposed method is considerably lower than that with the conventional method, resulting in reduced harmonic current content. Comparing Figure 8b,c with Figure 9b,c, the DC components of the harmonic currents in the respective coordinate systems, ${\hat{i}}_{d5\_dc}$ and ${\hat{i}}_{q5\_dc}$, are approximately 0.2 A and −0.15 A for the traditional strategy, and approximately 0.1 A and −0.05 A for the proposed strategy; similarly, ${\hat{i}}_{d7\_dc}$ and ${\hat{i}}_{q7\_dc}$ are approximately 0.25 A and −0.15 A for the traditional strategy, and approximately 0.1 A and −0.05 A for the proposed strategy. This indicates a reduction and stabilization in the DC components of the harmonic currents in each coordinate system. Through Fast Fourier Transform (FFT) and quantitative analysis of the fifth- and seventh-order stator current harmonics relative to the fundamental wave, with the horizontal axis representing harmonic order and the vertical axis representing the percentage of the fundamental wave, it can be seen from Figure 8d and Figure 9d that the proposed strategy reduces the fifth-order harmonic component by 2.2143% and the seventh-order harmonic component by 1.243%, with a total harmonic distortion (THD) reduction of 2.25%. This validates the effectiveness of the proposed strategy in suppressing fifth- and seventh-order stator current harmonics, effectively reducing these harmonics and enhancing the overall system stability.

**Figure 10:**
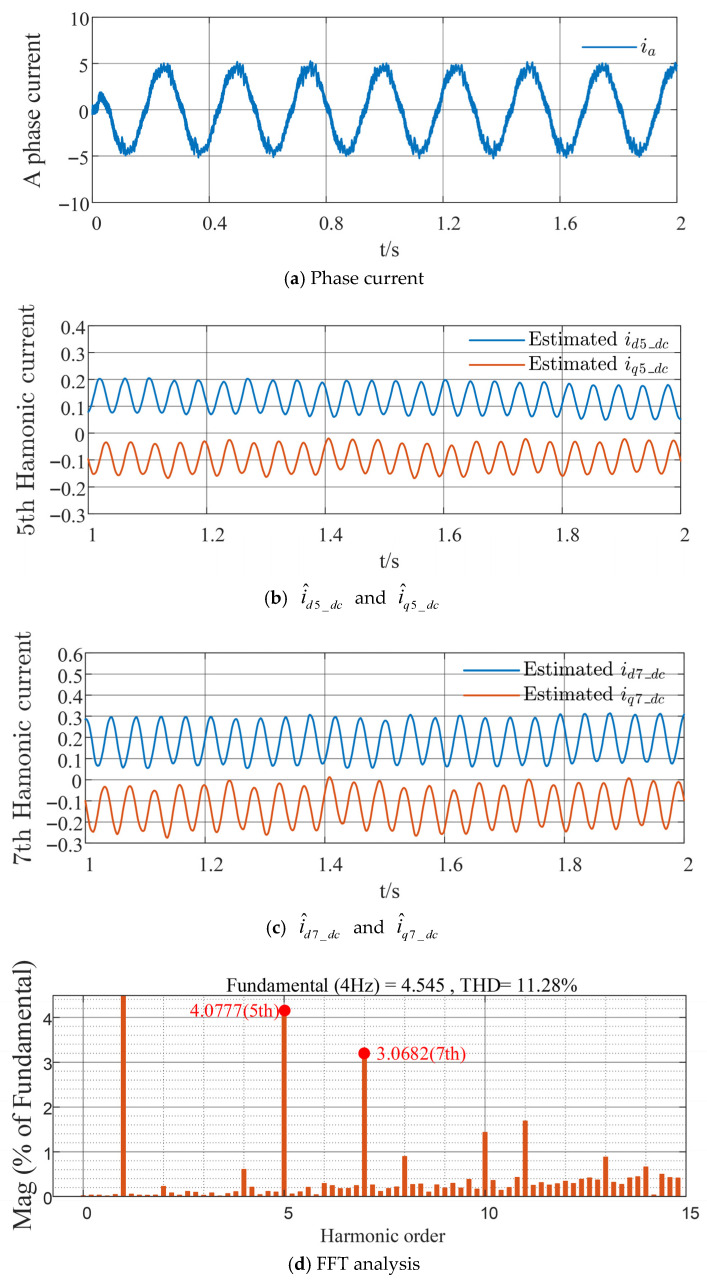
Experimental waveforms of the traditional HFSWI strategy at a given speed of 60 r/min and load torque of 5 N·m.

**Figure 11:**
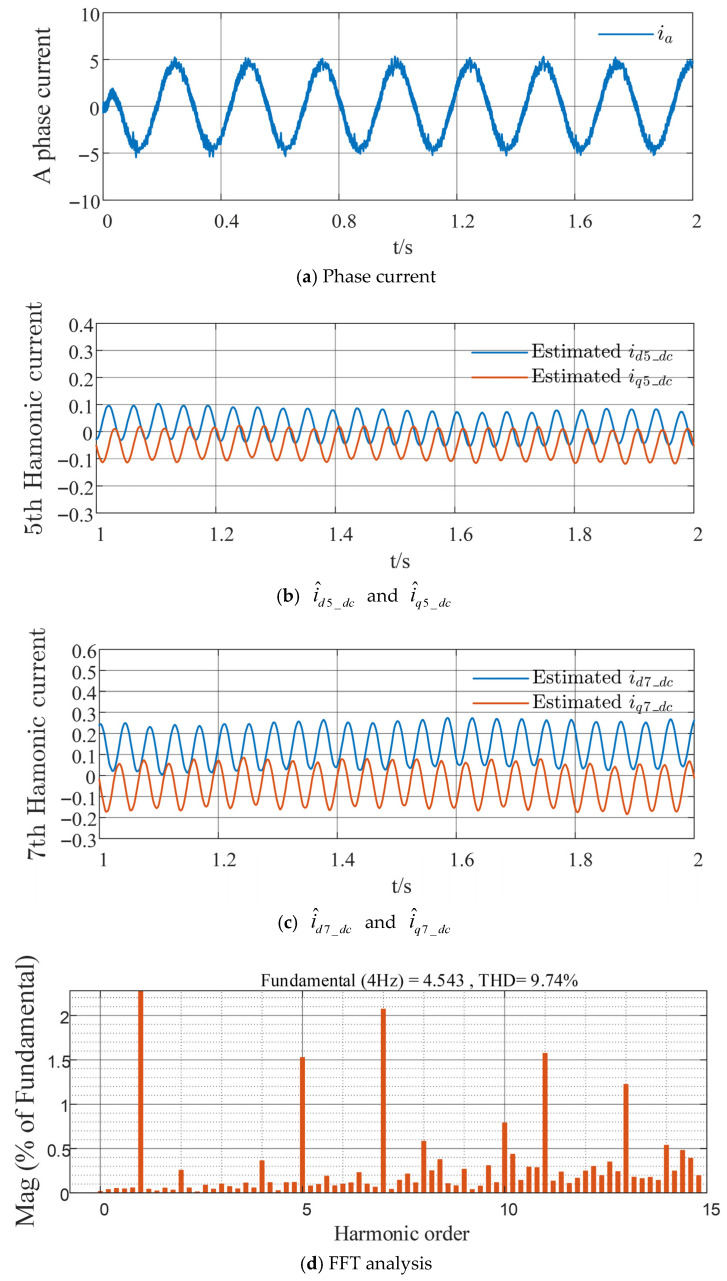
Experimental waveforms of the proposed strategy at a given speed of 60 r/min and load torque of 5 N·m.

Further verification is conducted on the harmonic suppression effectiveness of the proposed strategy at lower speeds and under different load conditions, as well as the resulting phase current distortion, as shown in Figure 10 and Figure 11. With the improved strategy, ${\hat{i}}_{d5\_dc}$ and ${\hat{i}}_{d7\_dc}$ decrease by 0.1 A and 0.05 A, respectively, whereas ${\hat{i}}_{q5\_dc}$ and ${\hat{i}}_{q7\_dc}$ increase by 0.05 A and 0.05 A respectively. The percentage contents of the fifth- and seventh-order harmonics are reduced by 2.5521% and 0.9969%, respectively. These results effectively validate the superiority of the proposed harmonic suppression strategy under various operating conditions.

**Figure 12:**
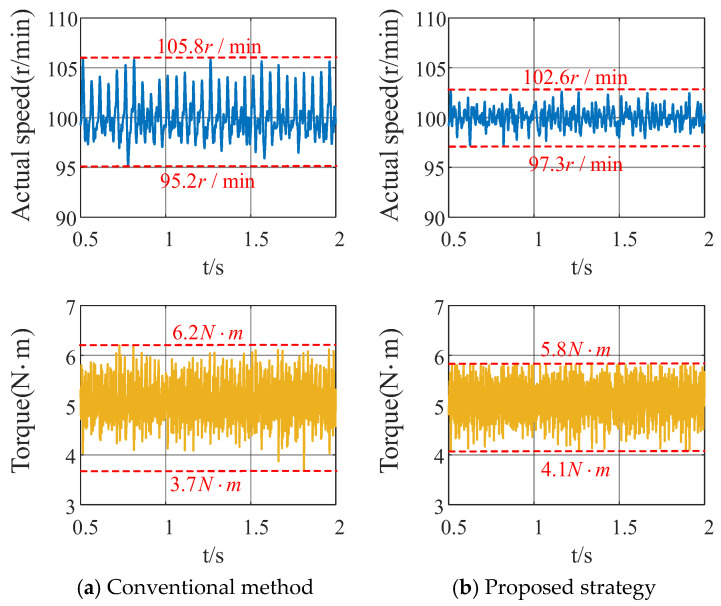
Speed and torque fluctuations of the traditional method and the proposed strategy at a given speed of 100 r/min and load torque of 5 N·m.

Subsequently, the fluctuations in speed and torque under the conditions of a given speed (100 r/min) and load torque (5 N·m) are analysed for both the conventional method and the proposed strategy, as shown in Figure 12. Comparing the actual speed fluctuations and torque fluctuations, respectively, the maximum speed fluctuation of the proposed strategy decreased by 3.2 r/min compared to the conventional strategy, while the minimum value increased by 2.1 r/min. Torque fluctuations were reduced overall by 0.8 N·m compared to the conventional strategy. This effectively demonstrates that attenuating the 5th and 7th stator current harmonics yields significant improvements in speed and torque fluctuations, achieving higher speed and torque control accuracy.

**Figure 13:**
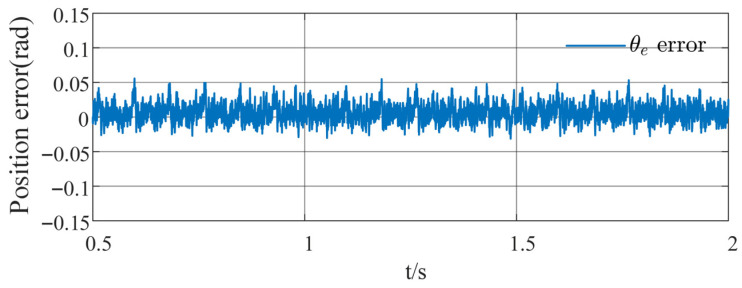
Rotor position error under a load torque of 5 N·m and a reference speed of 60 r/min.

As illustrated in Figure 13, the rotor position error waveform under a load torque of 5 N·m and a reference speed of 60 r/min exhibits positive and negative deviations within 0.06 rad. This confirms that the proposed strategy achieves high rotor position estimation accuracy.

## 6. Conclusions

This paper proposes a harmonic extraction and voltage compensation method based on a sixth-order proportional resonant controller for the sensorless control system of HFSWI in IPMSM. The method performs mathematical analysis on the introduced fundamental AC component and suppresses it using a proportional resonant controller, effectively reducing the content of fifth- and seventh-order stator current harmonics in the fundamental wave. The open-loop transfer function of the current loop is analyzed using Bode plots to determine the control parameters of the sixth-order proportional resonant controller. Simulation experiments conducted in MATLAB/Simulink, through comparisons of phase currents, harmonic currents, and FFT analysis, demonstrate that the proposed strategy achieves effective harmonic suppression, enhancing system stability. Finally, comparisons of actual speed and torque between the traditional method and the proposed strategy show that the proposed harmonic suppression strategy effectively reduces speed fluctuations and torque ripples.

## Figures and Tables

**Table 1:** Parameters of permanent magnet synchronous motor.

IPMSM Parameters	Value
Rated speed/(r·min^−1^)	1500
Stator resistance/Ω	0.958
Moment of inertia/(kg·m^2^)	0.003
d-axis inductance/mH	5.25
q-axis inductance/mH	12
Permanent magnet flux linkage/Wb	0.1827
Number of pole pairs	4

## Data Availability

Data are contained within the article.

## Abbreviations

The following abbreviations are used in this manuscript:

IPMSM	interior permanent magnet synchronous motor
HFSWI	high-frequency square-wave injection
QPR	quasi-proportional resonant
HF	high-frequency
EMF	electromotive force
HPF	high-pass filter
LPF	low-pass filter
PWM	pulse width modulation
DC	direct current
AC	alternating current
PI	proportional-integral
MTPA	maximum torque per ampere
FFT	fast Fourier transform
THD	total harmonic distortion
